# Disease-Induced Mortality Outweighs Hunting in Causing Wild Boar Population Crash After African Swine Fever Outbreak

**DOI:** 10.3389/fvets.2020.00378

**Published:** 2020-07-28

**Authors:** Kevin Morelle, Jakub Bubnicki, Marcin Churski, Jakub Gryz, Tomasz Podgórski, Dries P. J. Kuijper

**Affiliations:** ^1^Mammal Research Institute, Polish Academy of Sciences, Białowieza, Poland; ^2^Department of Game Management and Wildlife Biology, Faculty of Forestry and Wood Sciences, Czech University of Life Sciences, Prague, Czechia; ^3^Department of Forest Ecology, Forest Research Institute (IBL), Raszyn, Poland

**Keywords:** disease ecology, camera trap, culling strategies, host-disease interaction, *sus scrofa*

## Abstract

African swine fever (ASF) has been spreading in the Eurasian continent for more than 10 years now. Although the course of ASF in domestic pigs and its negative economic impact on the pork industry are well-known, we still lack a quantitative assessment of the impact of ASF on wild boar (*Sus scrofa*) populations under natural conditions. Wild boar is not only a reservoir for ASF; it is also one of the key wildlife species affecting structure and functioning of ecosystems. Therefore, knowledge on how ASF affects wild boar populations is crucial to better predict ecosystem response and for the design of scientific-based wild boar management to control ASF. We used a long-term camera trap survey (2012–2017) from the Białowieza Primeval Forest (BPF, Poland), where an ASF outbreak occurred in 2015, to investigate the impact of the disease on wild boar population dynamics under two contrasting management regimes (hunted vs. non-hunted). In the hunted part of BPF (“managed area”), hunting was drastically increased prior and after the first ASF case occurred (March 2015), whereas inside the National Park, hunting was not permitted (“unmanaged area,” first detected case in June 2015). Using a random encounter model (REM), we showed that the density and abundance of wild boar dropped by 84 and 95% within 1 year following ASF outbreak in the unmanaged and managed area, respectively. In the managed area, we showed that 11–22% additional mortality could be attributed to hunting. Our study suggests that ASF-induced mortality, by far, outweighs hunting-induced mortality in causing wild boar population decline and shows that intensified hunting in newly ASF-infected areas does not achieve much greater reduction of population size than what is already caused by the ASF virus.

## Introduction

In 2007, the African swine fever (ASF) virus reappeared in the Eurasian continent in Georgia (1, 2). From there, ASF further spread to the neighboring countries (3), entered the European Union in 2014 (4), and led most recently to local outbreaks in Western Europe (5, 6). Reported lethality rates induced by ASF were very high, reaching 95–100% in both domestic pigs and wild boar (7).

While concerns connected to this ASF outbreak focused mainly on threats to the pork industry and associated economic losses (8, 9), the impact of ASF on wild boar population size and the resulting consequences for ecosystem functioning has been so far neglected. Wild boar play a key role in the ASF cycle in Europe, facilitating virus transmission and survival in the environment (10). This wild boar–habitat cycle and its interaction with the domestic cycle is a major concern in Europe. Thus, understanding the impact of ASF on wild boar population is needed to better assess the dynamic of the wild boar–habitat transmission cycle.

To our knowledge, there are no published results on wild boar population mortality due to ASF under natural conditions. Considering that wild boar is one of the key species affecting structure and functioning of ecosystems globally (11–18), knowledge on how ASF affects wild boar populations is crucial to better predict ecosystem response and to gain knowledge to prepare a scientific-based wild boar management plan aimed to control ASF more effectively (19). The default policy in Europe consists in a drastic reduction of wild boar population before ASF incursion (20), and once the disease is present, an active carcass removal within the infected zone combined with intense hunting in buffered zones (21). However, host population and disease-management plans can interact and generate unexpected demographic and behavioral responses of the targeted populations (22, 23). In this respect, it is crucial to know the relative contribution of hunting actions and ASF in affecting wild boar population dynamics.

In this paper, we studied the dynamics of a wild boar population in the period 2012–2017 that overlapped with an ASF outbreak in 2015 in the Białowieza Primeval Forest (BPF, Poland). The BPF offers the unique opportunity to study wild boar population dynamics under two contrasting management regimes: a hunting-free area (“unmanaged area”) and an area with intensified wild boar culling in response to the ASF outbreak (“managed area”). We hypothesized that, in the managed area, wild boar population decline will be stronger and faster due to the additive impact of hunting- and ASF-induced mortality compared to the unmanaged area.

## Methods

### Study Area

The BPF, located in eastern Poland (52°450N, 23°500E) and western Belarus, is a large continuous forest composed of mixed deciduous stands. The BPF covers in total 1,450 km^2^ and consists of a mosaic of forest types, which is dominated by deciduous oak-lime-hornbeam forest. The climate is continental with a mean temperature of 6.8°C and a mean annual precipitation of 641 mm. Five native ungulate species occur in the BPF (in decreasing order of abundance): red deer (*Cervus elaphus*), wild boar, roe deer (*Capreolus capreolus*), European bison (*Bison bonasus*), and moose (*Alces alces*). These ungulate co-occur with two large carnivores: the Eurasian lynx (*Lynx lynx*) and the wolf (*Canis lupus*) (24). Before the ASF outbreak, wild boar belonged to the most abundant ungulate species both in numbers and in biomass (25). For a more detailed description of the study area, see (26).

**Figure 1:**
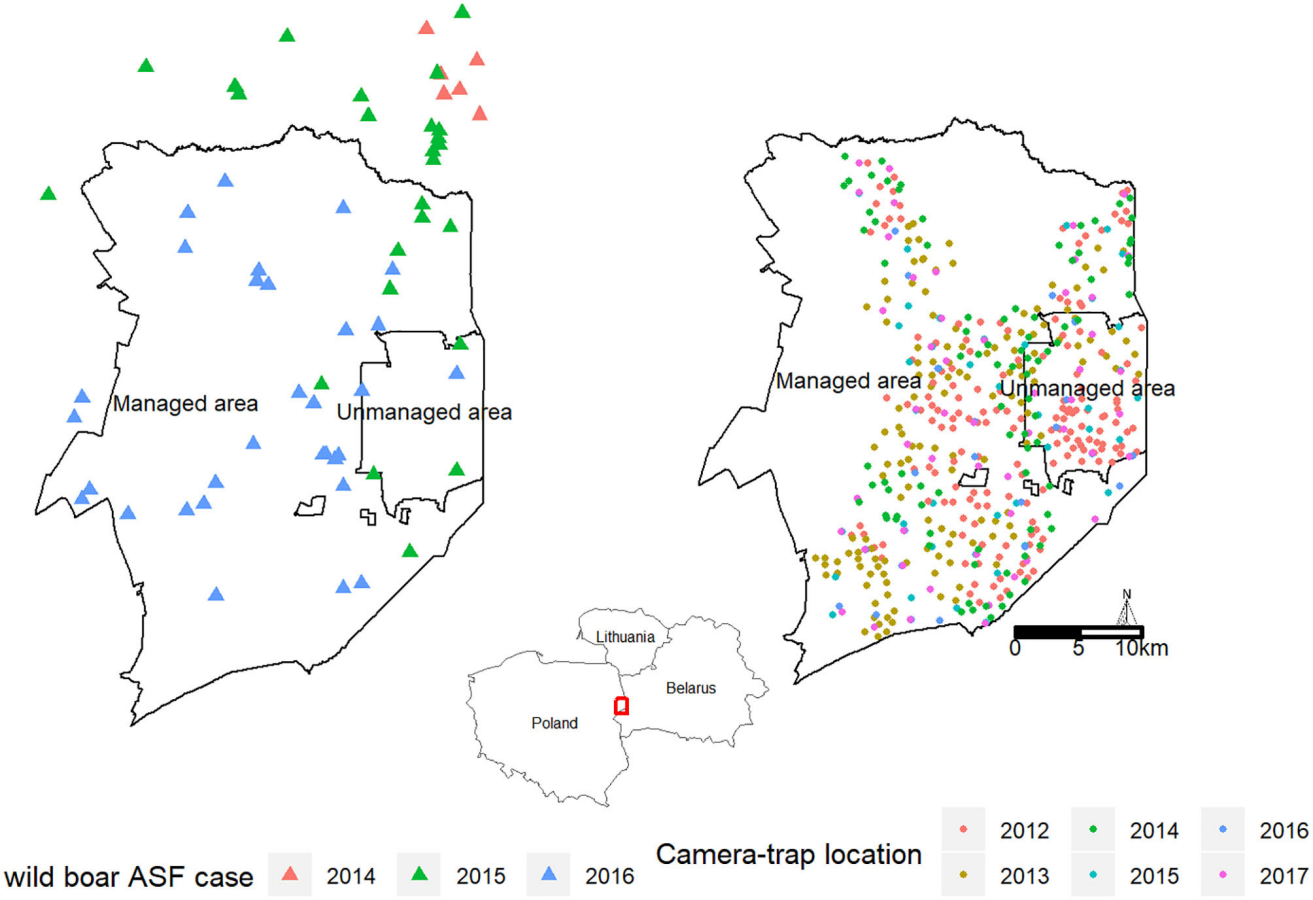
Study area, the Białowieza Primeval forest (BPF), with two management regimes. In the “managed area,” wild boar culling intensified as a result of the ASF outbreak, whereas, in the “unmanaged area,” no wildlife management actions were taken. Camera placement is indicated in different colors during the survey periods in 2012–2017.

In the polish part of the BPF, where our study was carried out, the area is divided into two management regimes (Figure 1). The largest protected part is the Białowieza National Park, which is managed for biodiversity conservation. Hunting is not allowed inside the national park (“unmanaged area,” area = 105 km^2^). The area outside the national park is managed for timber production (by the State Forest National Forest Holding), and ungulate numbers are regulated (“managed area,” area = 600 km^2^). Wild boar hunting is conducted all year round with the main hunting season occurring in winter (October–February).

**Figure 2:**
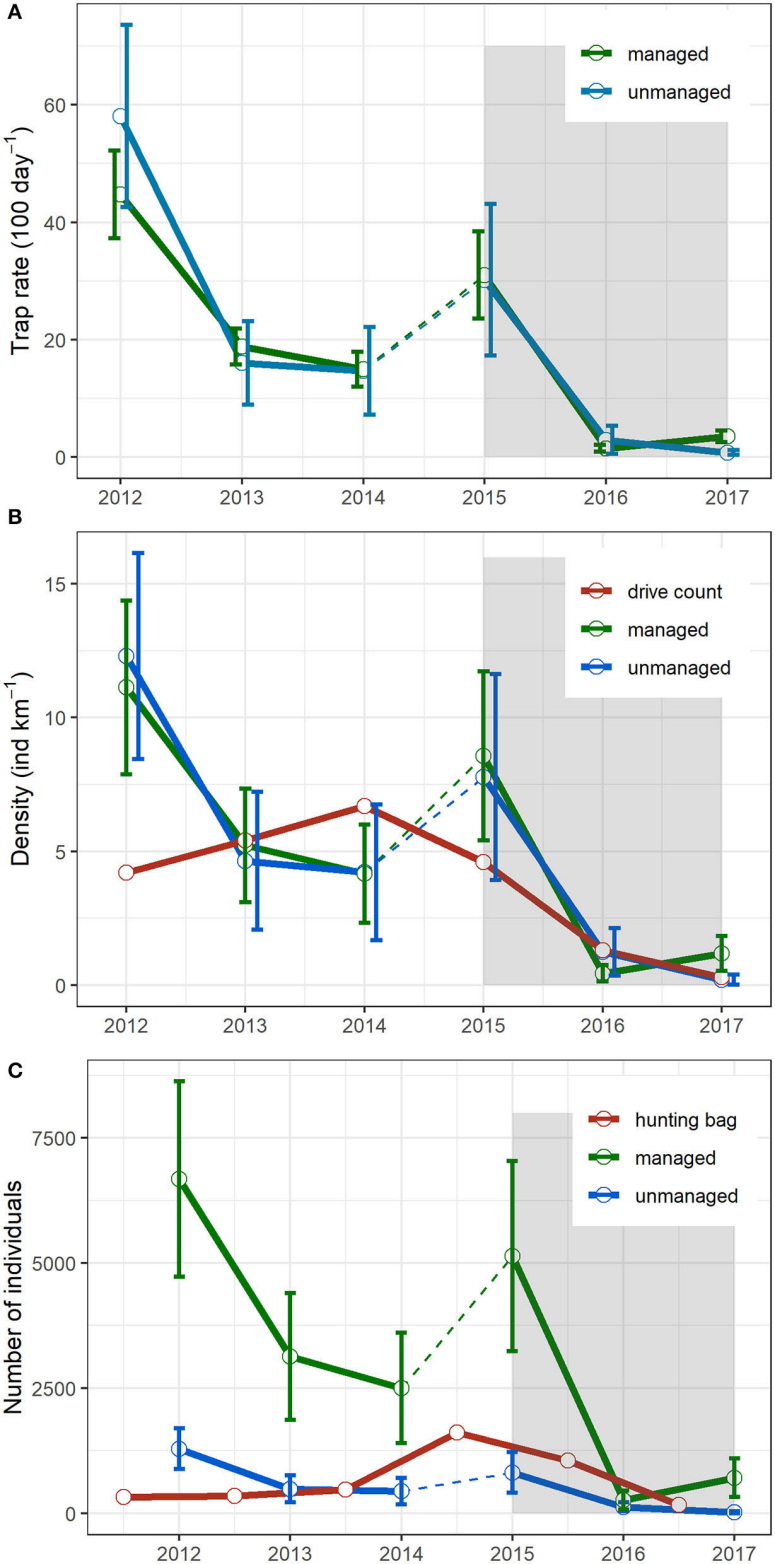
Wild boar trapping rates based on camera trap surveys **(A)**, wild boar density estimation based on the random encounter model and comparison with independent drive count estimates **(B)**, and derived abundance and comparison with hunting bag in the managed and the unmanaged parts of the BPF **(C)**. The dotted lines in between census years 2014 and 2015 indicate a change of camera trap placement. The shaded area represents the period where ASF is officially observed in the BPF.

In the region, the first cases of ASF in wild boar were detected in February 2014 near Sokółka in the northeastern part of the country at a distance of c. 50 km from the BPF (4), and the first official cases of ASF in the BPF were reported in March 2015 (Figure 1). In the managed parts of the BPF, hunting followed the national policy aimed at drastically reducing wild boar numbers prior to ASF arrival. This led to a 4-fold increase in hunting bags in 2014/2015 when compared to the average hunting bag over the 2005–2014 period (Figure 2C). In the following hunting season of 2015–2016, when the first case of ASF had been officially confirmed within the BPF, intense hunting actions continued (3-fold increase in hunting bag compared to 2005–2014). Inside the unmanaged area, no hunting or any other wild boar–targeted management actions took place in reaction to the ASF outbreak.

### Camera Trapping Design

We used available camera trap surveys taking place in the BPF between 2012 and 2017 to provide an objective estimate of wild boar population size. Because camera survey objectives varied over time, the study design (i.e., camera placement and timing) varied accordingly (Supplementary Figure 1). Specifically, between 2012 and 2014, camera traps followed a random placement design [see (26)] while between 2015 and 2017, cameras were placed along forest roads and trails to increase capture rates of large carnivores (26). We investigated the potential effect of this change in design (placement and timing) on wild boar population estimates in our analysis (see the section on *Detection Probability*). During the entire survey period, the same digital trail camera model (Ecotone SGN-5210A) was used. Cameras were triggered by passive infrared sensors with a detection angle of c. 35° and a maximal detection range of c. 20 m. After detection, with a time lag of 1 s, a photograph was taken and the camera recorded a 60-s video (26). During low-light conditions, cameras switched to a stealth infrared mode. Cameras were attached to a tree at a height of c. 1 m at locations with a clear view of at least 20 m [see (26)]. Camera trap surveys took place during summer and autumn (August–October), except for the 2014 survey (survey between January and March). Photographs and videos were manually analyzed and information on timestamp, the number of individuals, and when possible, age class (piglet, juvenile, or adult) and sex were recorded. The different camera trap surveys as well as the related data, photographs, and videos were managed using the open-source Trapper software (27).

### Data Analysis

#### Detection Probability

As the long-term wildlife monitoring in Białowieza contains changes in (i) camera trap placement (random vs. trail/road-based) and (ii) survey period (different seasons), we tested the effects of these two variables on the probability of detecting wild boar. To assess the impact of camera placement, we used the 2016 camera session, in which both methods of camera trap placement were used in a paired design. Specifically, 50 cameras were installed along the existing network of forest roads, and a paired camera was installed randomly ca. 200 m from the initial camera in the forest. To test for seasonal effects, we used the 2013 survey in which cameras were deployed continuously throughout the year (26). We pooled data on wild boar for each season (spring: March–May, summer: June–August, autumn: September–November, winter: December–February). We used a single-season occupancy model assigning the type of camera trap placement and the season as covariates (28). We used “camtrapR” (29) and “unmarked” packages (30) to prepare the dataset and to perform analysis within the R environment (31).

#### Camera Trapping Rate and Density Estimation

To quantify yearly changes in the wild boar population number, we used a relative index of abundance based on the wild boar trapping rates and a density estimate based on these figures. Camera trapping rate is defined as the ratio between the encounter rate, i.e., the total number of photographic events *y* and the camera trapping effort *t*, i.e., the number of 24-h periods each camera was deployed. To ensure independency between subsequent event records, we only used consecutive camera capture events (i.e., visiting individuals or groups of wild boar) with a minimum of 10-min interval between records (32). This resulted in the removal of 197 records from the full dataset comprising 2,089 records. For species, such as wild boar, that are difficult to individually recognize [but see (33)], methods considering the process of contact between animals and sensors have been developed. Here, specifically we used the random encounter model (REM), describing the rate of contact between moving animals and static cameras to estimate animal density (34). The REM requires information on the species number of encounter *y*, sampling effort (i.e., camera days) *t*, camera detection zone specified by radius *r* and angle *theta*, and an estimated average speed of movement of the target species *v*.

In case of social species like wild boar, individual records can be considered as group records, in which case REM density is multiplied by unbiased independent estimate of average group size *g* (34). Because camera trapping estimates are sensitive to group size (35) and ASF and culling pressure might have impacted wild boar group structure and size, we decided not to include this parameters in our REM. Our view is analogous to that put forward in the context of distance sampling of clustered animals. The authors of (36) acknowledge that treating grouped individuals as independent values may sometimes be necessary if accurate group counts are not easily obtained, or if groups are not cohesive, as is the case for lions (37). In this case, variance connected to the REM estimates will be inflated, but estimates remain unbiased (36). We thus calculated wild boar density (*D*) according to

$$\begin{array}{l} D=\frac{y}{t}\frac{\pi }{vr\left(2+\theta \right)} \end{array}$$

where *y* and *t* are the same as for the camera trap rate. Estimation of average speed *v* was based on daily range estimations from collared wild boar in the same study area (38). Considering the large underestimation of daily range movement with telemetry methods (39), we applied a correction factor to improve the daily range estimate following (40). Camera detection radius *r* was based on (41), and the angle *theta* was based on camera model specifications (Table 1). We estimated uncertainty around y/t using non-parametric bootstrapping (42), resampling camera trap locations with replacement 10,000 times (34).

**Table 1:** Parameters used in the random encounter model to estimate wild boar density.

**Parameters**	**Description**	**Value**	**References**
*y*	Number of independent photo-captures	–	This study
*t*	Camera effort (days)	–	This study
*v*	Daily range (km/day)	8.9 ± 3.4	Podgorski et al. (38)
*r*	Detection distance (km)	0.02	Bubnicki et al. (26)
*theta*	Detection angle (radian)	0.61	Bubnicki et al. (26)

Further, to account for uncertainty due to other parameters (*v* and *r*), we used the propagate package in R (43). Propagate uses first-/second-order Taylor approximation and Monte Carlo simulation to calculate uncertainty propagation. We ran 10,000 simulations of these variables using the mean and standard deviations obtained from our data for *y/t, v*, and *r*, fixing all the other parameters. We compared our estimates of population density derived from camera trap analyses to drive count estimates, the method applied in the BPF to assess ungulates population (25). Drive count consists of a yearly census organized in the same day (in February) in the whole BPF (both managed and unmanaged parts). During these drive count, more than 200 people (divided into mobile pushers and stationary observers placed at the compartment limits) counted animals in randomly selected forest compartments [see (25) for more details], covering 10% of the entire Białowieza forest (including the managed and unmanaged parts).

From our density estimates for the managed and unmanaged areas, we derived the total wild boar population size by multiplying by the study area size, i.e., 600 km^2^ for the managed and 105 km^2^ for the unmanaged area, respectively. Observed population decline was then calculated for the two areas as the relative change (in percent) in abundance between 2015 and 2016 survey. Variation around the population decline was estimated by taking the average between the maximal (i.e., mean+sd_abundance, 2015_ to mean-sd_abundance, 2016_ relation) and the minimal (i.e., mean-sd_abundance, 2015_ to mean+sd_abundance, 2016_ relation) possible decline.

Finally, to assess the relative impact of ASF- and hunting-induced mortality on wild boar population size, we used two approaches. In the first one, we simply compared the decrease in abundance between the managed and the unmanaged populations, assuming that (i) populations are closed and (ii) population growth is equal in the two areas, so that the difference in population decline between the areas can be attributed mainly to hunting. The population closure assumption is congruent with telemetry study indicating very few movements of individuals between managed and unmanaged areas (38). The assumption of similar population growth is also reasonable considering the comparable resource and climatic conditions occurring in the two adjacent areas. Specifically, we assumed that the observed population decline inside the unmanaged forest is only due to ASF, following

$$\begin{array}{r} decline_{unmanaged}=mortality_{ASF}=\\ \frac{abundance_{unmanaged,2016}-abundance_{unmanaged,2015}}{abundance_{unmanaged,2016}} \end{array}$$

Whereas, in the managed area, the total observed decline was due to both hunting and ASF.

$$\begin{array}{r} decline_{managed}=mortality_{ASF}+mortality_{hunting}=\\ \frac{abundance_{managed,2016}-abundance_{managed,2015}}{abundance_{managed,2016}} \end{array}$$

In the second approach, we focused on the managed area only, investigating the relative share of hunting- and ASF-induced mortality. Specifically, we calculated the contribution of hunting-induced mortality to the observed decline in wild boar population since the first case of ASF according to

$$\begin{array}{l} hunting_{percent}=\frac{hunting_{bag,2015-2016}}{abundance_{managed,2015}} \end{array}$$

In this calculation, we make the assumption that available figures of hunting bags are accurate, i.e., that all shot wild boar have been reported and no animals died after a hunting event following shot wounds (and thus were not reported).

## Results

Wild boar detection probability (i.e., probability of detecting wild boar) was not influenced (*t*-test, *t*_42_ = 3.15, *p* > 0.1) by camera placement (randomly placed 0.15 ± 0.05 sd vs. camera traps placed on roads 0.17 ± 0.05 sd) (Supplementary Figure 1). This result indicates that the change in camera placement that occurred during our study period unlikely affected our density estimates. Detection probability of wild boar differed between seasons (one-way ANOVA: *F*_3, 591_= 27.29, *p* < 0.001) with increasing wild boar detections from spring to autumn and a decline in winter (Supplementary Figures 2, 3). We therefore based our comparisons across years only on data collected in the same season, i.e., in summer–fall when wild boar numbers are highest for all years except 2014, for which only a winter survey was available. For the year 2014, we cautiously interpret the estimate when compared to other years.

Wild boar trapping rates in the managed and unmanaged areas followed the same pattern during the 2012–2017 survey period (Figure 2A and Supplementary Table 1). In both areas, the trapping rate decreased dramatically from 2015 onward and remained at a low level. Density estimates from the REM showed similar trends as the camera trapping rate. For both managed and unmanaged areas, the density dropped from 8.6 ± 3.2 (mean ± sd) and 7.8 ± 3.9 individuals km^−2^ in year 2015, to 0.4 ± 0.3 and 1.2 ± 0.9 individuals km^−2^ in year 2016, respectively (Figure 2B).

Comparing the population size between summer 2015 (just after the ASF outbreak in the BPF) and summer 2016, we observed a 94.8 ± 6.4% decline in the managed area and a 83.8 ± 25.5% decline in the unmanaged part (Figure 2C). This would indicate that hunting in the managed parts resulted in an 11% additional mortality to the ASF-induced mortality. When we compared hunting bags and abundance estimates of years 2015 and 2016 in the managed area, the relative share of hunting-induced mortality rose to 21.7 ± 11.2%, while ASF accounted for 78.3 ± 11.2%. This value is relatively close to the one observed for the unmanaged area (83.8% decline).

## Discussion

This is the first study to quantify the impact of ASF on the mortality of wild boar population under contrasting management conditions consisting of a hunted and a hunting-free area. After official presence of ASF within the borders of the BPF, we observed a population decline of 83.8 ± 25.5 and 94.8 ± 6.4% for the unmanaged and managed parts of this forest, respectively. This result only slightly corroborates our initial hypothesis that the wild boar population in the managed part will experience a stronger and faster decline due to the additive impact of hunting. Indeed, the observed difference (11%) between these two areas suggests that the intense hunting actions implemented during 2014–2015 and 2015–2016 had a relatively low additional impact on the observed population decline.

When investigating the relative hunting ASF share in the population decline using hunting bags and abundance estimates, we showed that the relative share of hunting- and ASF-induced mortality could be 21.7 and 78.3%, respectively. In our analysis, we assumed that hunting bag records are accurately reported and that all shot individuals have been retrieved and there is no additional delayed mortality following hunting events. Such underreporting of hunting bags could lead to an inaccuracy of the estimation of the hunting-induced mortality. Together, these two approaches suggest that ASF has a large impact on wild boar population, removing around 80% of the population in 1 year of disease presence. Furthermore, our results indicate that the increased hunting pressure during the ASF epidemic led to only a small additional impact on population decline.

Many lessons can be learned from the management actions implemented in the BPF in response to the ASF outbreak. The first management actions took place in 2014–2015 before the ASF presence in the BPF. It followed the Polish national emergency plan and EFSA recommendation to preventively reduce wild boar density before ASF introduction (44). To reach this aim, hunting pressure was increased dramatically (four-time increase in the number of wild boar shot compared to previous years' average) in the managed part of the BPF. The action apparently failed to reach its goal since the population density in the following year remained high (7.5 animals km^−2^), and no difference in trends between the managed and unmanaged parts could be observed (Figure 2B). This result is congruent with previous work demonstrating that wild boar population can still increase even when hunting mortality is increased (45). It further suggests that other environmental factors, such as climate (46) and pulsed resources (47), could have played a greater role in driving wild boar population dynamics than the increased intensity of hunting. High hunting pressure might also have induced unwanted effects inducing compensatory population growth rate and accelerated generation time, i.e., higher juvenile female contribution to the reproductive set (48) and earlier reproduction (49). In the managed part of the BPF, the camera trap data suggest such a positive feedback, with an increased ratio of observation of piglets and juveniles in the year following the hunting actions (unpublished result). The second action took place in 2015–2016 (a 3-fold increase in hunting bag compared to the years before the ASF outbreak) after the first case of ASF was already observed in the BPF and continued through 2016–2017. The second hunting action might have had an unwanted effect on the spread of ASF in the area itself and outside, i.e., increased transmission and large movement of groups and individual wild boar (44, 50).

In the BPF, ungulates drive counts are annually performed (see the *Methods* section). In general, the trend based on drive count density estimates was similar to the camera trap estimates (Figure 2B). But for some specific years (2012, 2014, and 2015), there were clear differences illustrating the inaccuracy of the population index approach like drive count census to capture population changes for the following reasons. The timing of the drive count, taking place in February before wild boar reproduction peak, does not allow seeing potential positive feedback of management actions on population dynamics (such as discussed above). Furthermore, drive count census provides a snapshot of the population status at one particular day (the day of the census) in a part of the area (10% of the study area), thus inaccurately taking into account existing spatiotemporal patterns in wild boar presence in the BPF [see (26)]. In comparison, camera trap surveys have been shown to be particularly efficient to monitor animal populations in various conditions (51). During the 2012–2017 survey period, we had a spatiotemporal coverage of the population of 0.07–0.50 camera km^−2^ deployed for a minimum of 3 months. The camera traps approach therefore provides a much more representative picture based on longer-term observations with a higher spatial resolution. We therefore argue that camera traps provide more reliable population size estimates, considering their higher spatial and temporal sampling resolution.

We are aware of some limitations of our study. Firstly, we assumed that only ASF and hunting influenced wild boar mortality. In the BNP, however, natural predators, lynx, and wolf are also present. The impact of these predators on wild boar is, however, moderate [predation from wolf and lynx has been estimated to account for 14% of mortality (24, 52)]. Since both wolf and lynx are not hunted in neither the managed nor the unmanaged area and both species occur in similar densities across the area (26), it is expected that predator-induced mortality rates are not largely different between the managed and unmanaged areas. Road casualties, another important cause of ungulate mortality, are not considered in our study. However, the road network in the BNP is very limited, and the number of casualties is negligible (25). Secondly, we used published parameters necessary for the computation of densities based on the REM. While daily range estimates come from the same study area, our density estimates would be improved if camera detection distance and angle parameters would be assessed specifically for our study.

Our study showed that the ASF outbreak led to a drop of 83.8 ± 25.5% and 94.8 ± 6.4% of the wild boar population in a non-hunted and a hunted area, respectively, within 1 year from the detection of the first ASF case. The observed wild boar decline was mostly due to ASF, and even a 3-fold increase in the hunting intensity during ASF outbreak had only minor additional effect (11–22%) on wild boar mortality in areas already affected by ASF. This fact has significant implications for management and disease control efforts. First, it appears reasonable to limit (or even ban) hunting activities in newly infected areas, at least during the first stages of epidemic, because the ASF virus appears to be more effective in reducing wild boar numbers, while intense hunting poses a high risk of virus spread, e.g., through fomites (53), disturbed animals (50), or hunters' movement (54). Effectiveness of such an approach is supported by its successful implementation in the Czech Republic and Belgium (55). Secondly, high ASF-induced mortality and subsequent abundance of infectious carcasses underline the critical importance of systematic carcass search and removal for effective disease control. This measure should help to reduce the viral load in the environment, enhance passive surveillance, and facilitate tracking of disease dynamics (56). To optimize resources use in ASF control, we suggest that hunting to reduce wild boar population size is reasonable only as a preemptive measure in anticipation of the disease and should be replaced by systematic carcass removal efforts once an epidemic breaks out.

While our results indicate that more than 80% of the wild boar population disappeared within 1 year of the ASF outbreak, one might wonder what happened with the remaining population. Do they get infected and recover, becoming carriers? The question has still no clear answer (57–59) but will need careful attention in post-infection areas (e.g., by means of hunted population surveillance) to ensure complete disease eradication. Another possibility is that the remaining population is made of individuals and/or groups of individuals that succeeded in avoiding the infection. In this case, we will need to know if there are specific traits favoring disease avoidance (e.g., age, sex, boldness)? These questions along with the relative impact of ASF on wild boar population structure and post-infection recovery will need careful attention in the coming time in order to improve our understanding of the ASF–wild boar system.

The drastic wild boar population decline observed in the BPF not only has important disease-management implications. It also has important implications in terms of ecosystem functioning, considering the fundamental roles played by wild boar (11–18). Pursuing monitoring of the population recovery along with forest dynamics will thus be of crucial importance in the coming years to better understand potential and so far unconsidered consequences of ASF on trophic cascades induced by wildlife diseases (60).

## Data Availability Statement

The datasets generated for this study are available on request to the corresponding author.

## Ethics Statement

Our study was not subject to permission/authorization from an ethical commission, since we used a non-invasive method (camera trapping) which does not disturb the natural behavior of animal.

## Author Contributions

KM, MC, DK, JB, and TP conceived the analysis. MC, DK, and JB designed and implemented the camera trap survey and the data collection. KM performed data analysis and wrote the first manuscript draft. All authors discussed the results and contributed to the final manuscript.

## Conflict of Interest

The authors declare that the research was conducted in the absence of any commercial or financial relationships that could be construed as a potential conflict of interest.

## References

[1] Rowlands RJ Michaud V Heath L Hutchings G Oura C Vosloo W. African swine fever virus isolate, Georgia, 2007. Emerg Infect Dis. (2008) 14:1870–4. 10.3201/eid1412.080591 19046509 PMC2634662

[2] Sánchez-Vizcaíno JM Mur L Martínez-López B. African swine fever (ASF): five years around Europe. Vet Microbiol. (2013) 165:45–50. 10.1016/j.vetmic.2012.11.030 23265248

[3] Vergne T Gogin A Pfeiffer DU. Statistical exploration of local transmission routes for African swine fever in pigs in the Russian federation, 2007–2014. Transb Emerg Dis. (2015) 64:504–12. 10.1111/tbed.12391 26192820

[4] Smietanka K Wozniakowski G Kozak E Niemczuk K Fraczyk M Bocian Ł. African swine fever epidemic, Poland, 2014–2015. Emerg Infect Dis. (2016) 22:1201–7. 10.3201/eid2207.151708 27314611 PMC4918169

[5] Chenais E Depner K Guberti V Dietze K Viltrop A Ståhl K. Epidemiological considerations on African swine fever in Europe 2014–2018. Porc Health Manag. (2019) 5:6. 10.1186/s40813-018-0109-2 30637117 PMC6325717

[6] Linden A Licoppe A Volpe R Paternostre J Lesenfants C Cassart D. Summer 2018: African swine fever virus hits north-western Europe. Transb Emerg Dis. (2019) 66:54–55. 10.1111/tbed.13047 30383329

[7] Gallardo MC Reoyo A de la T Fernández-Pinero J Iglesias I Muñoz MJ Arias ML. African swine fever: a global view of the current challenge. Porc Health Manag. (2015) 1:21. 10.1186/s40813-015-0013-y 28405426 PMC5382474

[8] Andraud M Halasa T Boklund A Rose N. Threat to the french swine industry of African swine fever: surveillance, spread, and control perspectives. Front Vet Sci. (2019) 6:248. 10.3389/fvets.2019.00248 31417915 PMC6681701

[9] Sánchez-Cordón PJ Montoya M Reis AL Dixon LK. African swine fever: A re-emerging viral disease threatening the global pig industry. Vet J. (2018) 233:41–48. 10.1016/j.tvjl.2017.12.025 29486878 PMC5844645

[10] Chenais E Ståhl K Guberti V Depner K. Identification of wild boar-habitat epidemiologic cycle in african swine fever epizootic. Emerg Infect Dis. (2018) 24:810–2. 10.3201/eid2404.172127 29553337 PMC5875284

[11] Burrascano S Copiz R Del Vico E Fagiani S Giarrizzo E Mei M Wild boar rooting intensity determines shifts in understorey composition and functional traits. Commun Ecol. (2015) 16:244–53. 10.1556/168.2015.16.2.12

[12] Cocquelet A Mårell A Bonthoux S Baltzinger C Archaux F Direct and indirect effects of ungulates on forest birds' nesting failure? An experimental test with artificial nests. For Ecol Manag. (2019) 437:148–55. 10.1016/j.foreco.2019.01.025

[13] Gómez JM Hódar JA Wild boars (Sus scrofa) affect the recruitment rate and spatial distribution of holm oak (Quercus ilex). For Ecol Manag. (2008) 256:1384–9. 10.1016/j.foreco.2008.06.045

[14] Heinken T Schmidt M Oheimb G von Kriebitzsch W-U Ellenberg H Soil seed banks near rubbing trees indicate dispersal of plant species into forests by wild boar. Basic Appl Ecol. (2006) 7:31–44. 10.1016/j.baae.2005.04.006

[15] Jaroszewicz B Piroznikow E Sondej I Endozoochory by the guild of ungulates in Europe's primeval forest. For Ecol Manag. (2013) 305:21–8. 10.1016/j.foreco.2013.05.004

[16] Sandom CJ Hughes J Macdonald DW Rewilding the scottish highlands: do wild boar, sus scrofa, use a suitable foraging strategy to be effective ecosystem engineers? Restor Ecol. (2013) 21:336–43. 10.1111/j.1526-100X.2012.00903.x

[17] Sondej I Kwiatkowska-Falinska AJ Effects of wild boar (sus scrofa L.) rooting on seedling emergence in białowieza forest. Pol J Ecol. (2017) 65:380–9. 10.3161/15052249PJE2017.65.4.007

[18] Welander J Spatial and temporal dynamics of wild boar (Sus scrofa) rooting in a mosaic landscape. J Zool. (2000) 252:263–71. 10.1111/j.1469-7998.2000.tb00621.x

[19] Vicente J Apollonio M Blanco-Aguiar JA Borowik T Brivio F Casaer J. Science-based wildlife disease response. Science. (2019) 364:943–4. 10.1126/science.aax4310 31171687

[20] Schulz K Conraths FJ Blome S Staubach C Sauter-Louis C African swine fever: fast and furious or slow and steady? Viruses. (2019) 11:866 10.3390/v11090866 PMC6783890 31533266

[21] Boklund A Cay B Depner K Földi Z Guberti V Masiulis M. Epidemiological analyses of African swine fever in the European union (November 2017 until November 2018). EFSA J. (2018) 16:e05494. 10.2903/j.efsa.2018.5494 32625771 PMC7009685

[22] Choisy M Rohani P. Harvesting can increase severity of wildlife disease epidemics. Proc Biol Sci. (2006) 273:2025–34. 10.1098/rspb.2006.3554 16846909 PMC1635483

[23] Streicker DG Recuenco S Valderrama W Gomez Benavides J Vargas I Pacheco V. Ecological and anthropogenic drivers of rabies exposure in vampire bats: implications for transmission and control. Proc Roy Soc B Biol Sci. (2012) 279:3384–92. 10.1098/rspb.2012.0538 22696521 PMC3396893

[24] Jedrzejewski W Schmidt K Theuerkauf J Jedrzejewska B Selva N Zub K Kill rates and predation by wolves on ungulate populations in białowieza primeval forest (Poland). Ecology. (2002) 83:1341–56. 10.2307/3071948

[25] Jedrzejewska B Jedrzejewski W Bunevich AN Milkowski L Krasinski ZA Factors shaping population densities and increase rates of ungulates in bialowieza primeval forest (Poland and Belarus) in the 19th and 20th centuries. Acta Theriol. (1997) 42:399–451.

[26] Bubnicki JW Churski M Schmidt K Diserens TA Kuijper DP. Linking spatial patterns of terrestrial herbivore community structure to trophic interactions. eLife. (2019) 8:e44937. 10.7554/eLife.44937.062 31577225 PMC6805123

[27] Bubnicki JW Churski M Kuijper DPJ Trapper: an open source web-based application to manage camera trapping projects. Methods Ecol Evol. (2016) 7:1209–16. 10.1111/2041-210X.12571

[28] MacKenzie ND I. *Occupancy Estimation and Modeling*. Inferring Patterns and Dynamics of Species Occurrence. Burlington, MA: Academic Press (2006).

[29] Niedballa J Sollmann R Courtiol A Wilting A camtrapR: An R package for efficient camera trap data management. Methods Ecol Evol. (2016) 7:1457–62. 10.1111/2041-210X.12600

[30] Fiske I Chandler R Unmarked: an r package for fitting hierarchical models of wildlife occurrence and abundance. J Stat Softw. (2011) 43:1–23. 10.18637/jss.v043.i10

[31] R Core Team R: A language and environment for statistical computing. Vienna: R Foundation for Statistical Computing (2019)

[32] Massei G Coats J Lambert MS Pietravalle S Gill R Cowan D. Camera traps and activity signs to estimate wild boar density and derive abundance indices. Pest Manag Sci. (2018) 74:853–60. 10.1002/ps.4763 29024317

[33] Hebeisen C Fattebert J Baubet E Fischer C Estimating wild boar (Sus scrofa) abundance and density using capture–resights in canton of Geneva, Switzerland. Eur J Wildlife Res. (2008) 54:391–401. 10.1007/s10344-007-0156-5

[34] Rowcliffe JM Field J Turvey ST Carbone C. Estimating animal density using camera traps without the need for individual recognition. J Appl Ecol. (2008) 45:1228–36. 10.1111/j.1365-2664.2008.01473.x26640297

[35] Chauvenet ALM Gill RMA Smith GC Ward AI Massei G Quantifying the bias in density estimated from distance sampling and camera trapping of unmarked individuals. Ecol Model. (2017) 350:79–86. 10.1016/j.ecolmodel.2017.02.007

[36] Thomas L Buckland ST Rexstad EA Laake JL Strindberg S Hedley SL. Distance software: design and analysis of distance sampling surveys for estimating population size. J Appl Ecol. (2010) 47:5–14. 10.1111/j.1365-2664.2009.01737.x 20383262 PMC2847204

[37] Cusack JJ Dickman AJ Rowcliffe JM Carbone C Macdonald DW Coulson T. Random versus game trail-based camera trap placement strategy for monitoring terrestrial mammal communities. PLoS ONE. (2015) 10:e126373. 10.1371/journal.pone.0126373 25950183 PMC4423779

[38] Podgórski T Baś G Jedrzejewska B Sönnichsen L Sniezko S Jedrzejewski W Okarma H Spatiotemporal behavioral plasticity of wild boar (*Sus scrofa*) under contrasting conditions of human pressure: primeval forest and metropolitan area. J Mammal. (2013) 94:109–19. 10.1644/12-MAMM-A-038.1

[39] Rowcliffe JM Jansen PA Kays R Kranstauber B Carbone C Wildlife speed cameras: measuring animal travel speed and day range using camera traps. Remote Sens Ecol Conserv. (2016) 2:84–94. 10.1002/rse2.17

[40] Palencia P Vicente J Barroso P Barasona JÁ Soriguer RC Acevedo P Estimating day range from camera-trap data: the animals' behaviour as a key parameter. J Zool. (2019) 309:182–90. 10.1111/jzo.12710

[41] Hofmeester TR Rowcliffe JM Jansen PA A simple method for estimating the effective detection distance of camera traps. Remote Sens Ecol Conserv. (2017) 3:81–9. 10.1002/rse2.25

[42] Efron B Tibshirani R Bootstrap methods for standard errors, confidence intervals, and other measures of statistical accuracy. Stat Sci. (1986) 1:54–75. 10.1214/ss/1177013815

[43] Spiess A-N. *Propagate: Propagation of* Uncertainty. (2018). Available online at: https://CRAN.R-project.org/package=propagate (accessed May 21, 2020).

[44] European Food Safety Authority Evaluation of possible mitigation measures to prevent introduction and spread of African swine fever virus through wild boar. EFSA Jl. (2014) 12:3616 10.2903/j.efsa.2014.3616

[45] Toïgo C Servanty S Gaillard J-M Brandt S Baubet E Disentangling natural from hunting mortality in an intensively hunted wild boar population. J Wildlife Manag. (2008) 72:1532–9. 10.2193/2007-378

[46] Vetter SG Ruf T Bieber C Arnold W. What is a mild winter? Regional differences in within-species responses to climate change. PLoS ONE. (2015) 10:e0132178. 10.1371/journal.pone.0132178 26158846 PMC4497731

[47] Servanty S Gaillard J-M Toigo C Brandt S Baubet E. Pulsed resources and climate-induced variation in the reproductive traits of wild boar under high hunting pressure. J Anim Ecol. (2009) 78:1278–90. 10.1111/j.1365-2656.2009.01579.x 19549145

[48] Servanty S Gaillard J-M Ronchi F Focardi S Baubet É Gimenez O Influence of harvesting pressure on demographic tactics: Implications for wildlife management. J Appl Ecol. (2011) 48:835–43. 10.1111/j.1365-2664.2011.02017.x

[49] Gamelon M Besnard A Gaillard J-M Servanty S Baubet E Brandt S. High hunting pressure selects for earlier birth date: wild boar as a case study. Evolution. (2011) 65:3100–12. 10.1111/j.1558-5646.2011.01366.x 22023578

[50] Scillitani L Monaco A Toso S Do intensive drive hunts affect wild boar (*Sus scrofa*) spatial behaviour in Italy? Some evidences and management implications. Eur J Wildlife Res. (2009) 56:307–18. 10.1007/s10344-009-0314-z

[51] Wearn OR Glover-Kapfer P. Snap happy: camera traps are an effective sampling tool when compared with alternative methods. Roy Soc Open Sci. (2019) 6:181748. 10.1098/rsos.181748 31032031 PMC6458413

[52] Jedrzejewska B Jedrzejewski W Predation in Vertebrate Communities: The Bialowieza Primeval Forest as a Case Study. Berlin: Springer-Verlag (1998).

[53] Mazur-Panasiuk N Zmudzki J Wozniakowski G. African swine fever virus – persistence in different environmental conditions and the possibility of its indirect transmission. J Vet Res. (2019) 63:303–10. 10.2478/jvetres-2019-0058 31572808 PMC6749736

[54] Mysterud A Rivrud IM Gundersen V Rolandsen CM Viljugrein H. The unique spatial ecology of human hunters. Nat Hum Behav. (2020) 10.1038/s41562-020-0836-7 32203320

[55] Miteva A Papanikolaou A Gogin A Boklund A Bøtner A Linden A. Epidemiological analyses of african swine fever in the european union (november 2018 to october 2019). EFSA J. (2020) 18:e05996. 10.2903/j.efsa.2020.599632625771

[56] Marcon A Linden A Satran P Gervasi V Licoppe A Guberti V. R0 estimation for the African swine fever epidemics in wild boar of czech republic and Belgium. Vet Sci. (2020) 7:2. 10.3390/vetsci7010002 31892104 PMC7157672

[57] Eblé PL Hagenaars TJ Weesendorp E Quak S Moonen-Leusen HW Loeffen WLA. Transmission of African swine fever virus via carrier (survivor) pigs does occur. Vet Microbiol. (2019) 237:108345. 10.1016/j.vetmic.2019.06.018 31521391

[58] Petrov A Forth JH Zani L Beer M Blome S. No evidence for long-term carrier status of pigs after African swine fever virus infection. Transb Emerg Dis. (2018) 65:1318–28. 10.1111/tbed.12881 29679458

[59] Ståhl K Sternberg-Lewerin S Blome S Viltrop A Penrith M-L Chenais E. Lack of evidence for long term carriers of African swine fever virus - a systematic review. Virus Res. (2019) 272:197725. 10.1016/j.virusres.2019.197725 31430503

[60] Buck JC Ripple WJ. Infectious agents trigger trophic cascades. Trends Ecol Evol. (2017) 32:681–94. 10.1016/j.tree.2017.06.009 28736043

