# Supplementary material for: Disease-Induced Mortality Outweighs Hunting in Causing Wild Boar Population Crash After African Swine Fever Outbreak
Source: Front Vet Sci. 2020 Jul 28;7:378. doi: 10.3389/fvets.2020.00378 (PMC7399055; doi:10.3389/fvets.2020.00378)
Supplement: Supplementary file 1 [file Data_Sheet_1.docx]

Supplementary Material

# Supplementary data


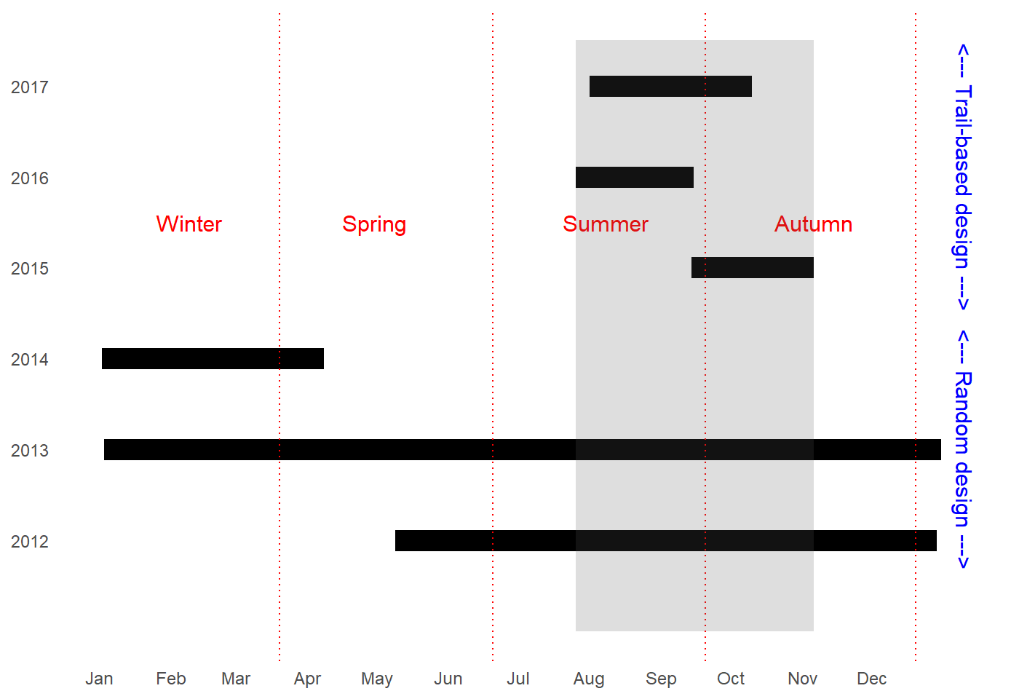


Supplementary Figure 1. Temporal coverage and associated design of the camera trap survey in BPF between 2012 and 2017. In grey is the indicated the period considered for analysis.

**Supplementary Table 1**. Camera trap effort and rate by year and study area.

| year | NP | # camera | trap coverage (/km²) | # camera days (t) | # of events (y) | trap rate (/100 camera days, (~event=group)) | trap rate (~event=ind) |
| --- | --- | --- | --- | --- | --- | --- | --- |
| 2012 | unmanaged | 54 | 0.51 | 752 days | 134 | 17.8 | 53.5 |
|  | managed | 113 | 0.19 | 1432 days | 199 | 13.9 | 43.9 |
| 2013 | unmanaged | 17 | 0.16 | 178 days | 18 | 10.1 | 16.9 |
|  | managed | 122 | 0.20 | 1491 days | 141 | 9.5 | 19.05 |
| 2014 | unmanaged | 11 | 0.10 | 120 days | 14 | 11.7 | 15.0 |
|  | managed | 76 | 0.13 | 832 days | 67 | 8.1 | 15.1 |
| 2015 | unmanaged | 15 | 0.14 | 700 days | 118 | 16.9 | 29.6 |
|  | managed | 57 | 0.10 | 2550 days | 329 | 12.9 | 32.4 |
| 2016 | unmanaged | 9 | 0.09 | 350 days | 3 | 0.9 | 4.3 |
|  | managed | 42 | 0.07 | 1595 days | 12 | 0.8 | 1.6 |
| 2017 | unmanaged | 9 | 0.09 | 429 days | 3 | 0.7 | 70 |
|  | managed | 42 | 0.07 | 1907 days | 26 | 1.4 | 4.2 |

# Supplementary analysis

## 2.1 Detection probability

The aim of this section is to investigate whether camera placement and season affect probability of detecting wild boar in Bialowieza forest, which are two issues (change in the survey design and survey timing) inherent to the dataset used. This step is crucial to know whether the comparison of abundance estimates between years will be plausible. The first constrain of the dataset is due to the change in design that took place in 2015 and which is due to the change in camera trapping project (from ungulate to carnivore monitoring). For the carnivore project, camera where place along the road in order to increase detection rate of targeted predators (lynx, wolves). The second constraint takes place in 2013 where the camera survey took mostly place during Winter, which is a different timing than for other year (Figure 1). This is an important step to know whether data from the random and the road/trail-based design can be compared. We used the single-season occupancy model assigning the type of plot (road vs. forest) as a site covariates. Datasets were prepared with the camtrapR package (for detection histories and camera operability dataframe) and analysis were made with unmarked package using the occu function.

### Camera placement effect

Our results showed no difference (t-test, t_(42)_=3.15, p > 0.1) in detection probability between camera along trail/road network and in forest (detection = 0.17±0.05 SE along road vs. 0.15±0.05 SE when camera placed in forest).


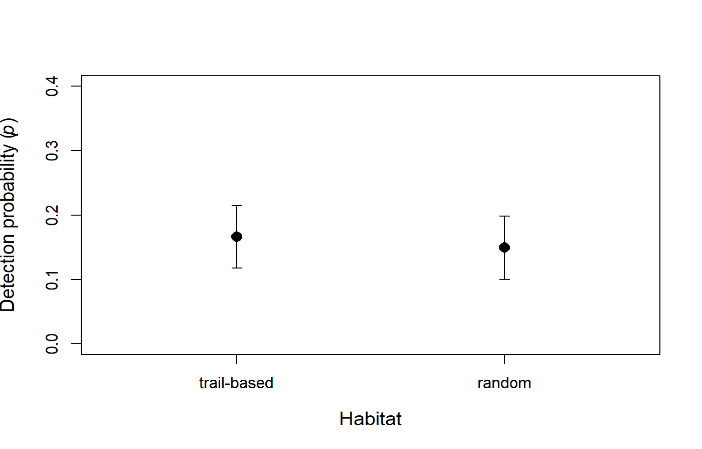


**Supplementary Figure 2**.Detection probability per plot type (trail-based vs. random).

### Seasonal effect

At the seasonal level, the one-way ANOVA test was significant (F_(3,591)_=27.29, p > .001) indicating difference in detection probability between seasons.


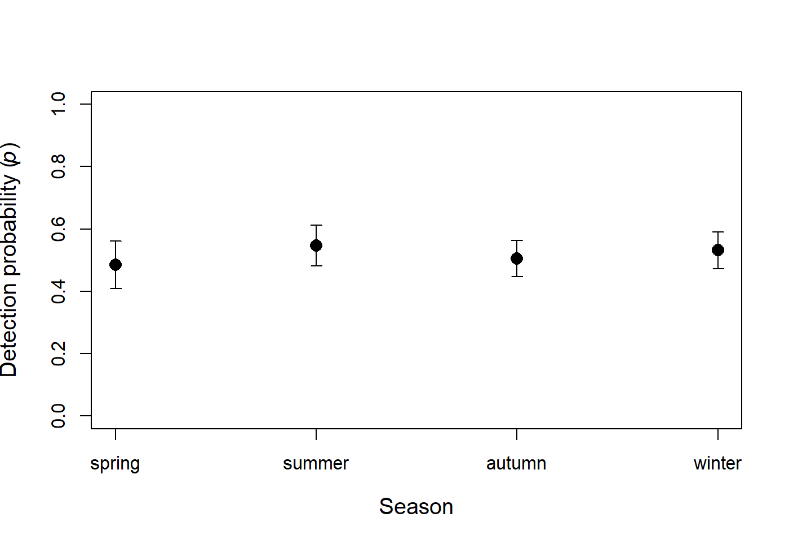


**Supplementary Figure 3**.Detection probability per season.

The pairwise comparison using Tukey Honest Significant Differences showed that all paired seasons differed apart from winter-summer and winter-fall Supplementat.

**Supplementary Table 2**. Pairwise comparison using Tukey Honest Significant Differences indicatedthat all paired seasons differed apart from spring and autumn.

| comparison | estimate | conf.low | conf.high | adj.p.value |
| --- | --- | --- | --- | --- |
| summer-spring | 0.06266348 | 0.04241573 | 0.08291123 | 4.90E+04 |
| fall-spring | 0.01090368 | -0.00836347 | 0.03017082 | 0.46372672 |
| winter-spring | 0.0379109 | 0.0165759 | 0.0592459 | 3.39E+09 |
| fall-summer | -0.0517598 | -0.06827351 | -0.03524609 | 4.90E+04 |
| winter-summer | -0.02475258 | -0.04363807 | -0.00586708 | 0.00433731 |
